# Long-Term Physicochemical Stability of Silver and Selenium Nanoparticles Designed for Caries Arrest: A 12-Month Evaluation

**DOI:** 10.3390/nano16211365

**Published:** 2026-10-26

**Authors:** Amjad Almuqrin, Chaminda Jayampath Seneviratne, Laurence J. Walsh, Sobia Zafar

**Affiliations:** 1 School of Dentistry, The University of Hail, Hail 2440, Saudi Arabia; a.almuqrin@uq.edu.au; 2 School of Dentistry, The University of Queensland, Brisbane, QLD 4006, Australia; jaya.seneviratne@uq.edu.au (C.J.S.); l.walsh@uq.edu.au (L.J.W.)

**Keywords:** silver nanoparticles, selenium nanoparticles, capping agents, shelf-life, colloidal stability, caries arrest, aggregation, agglomeration

## Abstract

***Purpose*:** To evaluate the 12-month physicochemical stability of silver and selenium nanoparticles (NPs) fabricated initially for topical caries arrest. ***Materials and Methods*:** The nanoparticles were synthesised via chemical reduction and capped with naturally derived agents. Post-synthesis suspensions were washed and stored at 4 °C in the dark for 12 months. Stability was assessed by comparing the physicochemical properties at baseline and 12 months. The characterisation approaches included Tyndall scattering and UV–visible spectroscopy, transmission electron microscopy, and inductively coupled plasma optical emission spectroscopy. ***Results*:** As formulations aged, they showed differences in stability. Citrate-capped silver NPs (AgL1) retained a defined surface plasmon resonance and showed a 6.7% reduction in elemental concentration. Silver NPs capped with casein (Ag1) and bovine serum albumin (Ag2) showed moderate reductions in elemental concentration (26.9% and 54.8%, respectively), accompanied by the formation of larger particle populations (~20–40 nm). Their initial mean particle sizes were 3.6 ± 3.4 nm for Ag1 and 2.5 ± 0.8 nm for Ag2. In contrast, silver NPs capped with citrate and tannic acid (AgL2) exhibited a substantial reduction in elemental concentration (88.6%), accompanied by pronounced particle aggregation. Selenium nanoparticles were the least stable: UV–Vis absorption features collapsed, TEM showed agglomeration (>70–100 nm), and ICP–OES indicated a significant reduction in the measured elemental concentration in the analysed suspension especially in Se1 (99.9%) and Se2 (99.96%). ***Conclusions*:** Under refrigerated, dark storage for 12 months, citrate-capped silver nanoparticles showed preserved integrity, whereas others showed moderate to severe issues with colloidal and elemental stability.

## 1. Introduction

The antimicrobial performance, safety, and translational potential of nanoparticles (NPs) in biomedical applications are largely defined by their long-term stability [1]. The antimicrobial activity of metallic and metalloid NPs can change over time owing to changes in their physicochemical properties [2]. Deterioration may manifest as aggregation/agglomeration, oxidation/reduction, dissolution or sedimentation, and is reflected in alterations to particle size, morphology, surface characteristics, surface charge and ion release. For instance, silver NPs (AgNPs) tend to oxidise to silver oxide, or aggregate in aqueous and biological media within days to weeks [3]. Similarly, selenium NPs (SeNPs) may transform into selenite compounds or bulk selenium when unstable, which undermines their functional properties [4]. Therefore, achieving and maintaining colloidal stability is fundamental to preserving the integrity of NPs. This can be accomplished through three key aspects: controlled synthesis, appropriate capping agents and standardised storage conditions, which can ensure reproducible and sustainable biological reactivity and enable reliable incorporation into clinical formulations. Optimised NP fabrication is the first step towards longevity, as it determines NP dispersion [5], nucleation [6], and surface charge [7]. Controlled reaction kinetics ensure a homogeneous particle size and morphology, which minimise the surface energy changes that promote aggregation [8]. Moreover, consistent nucleation inhibits Ostwald ripening, thereby maintaining colloidal uniformity [6]. In chemical synthesis, precise adjustment of precursor ratios [9], temperature [10], and reducing conditions [11] facilitates complete reduction.

Likewise, the timely introduction of stabilising agents enables dense capping layers that provide steric and electrostatic repulsion between particles [12]. In contrast, poorly regulated synthesis processes generate polydisperse, partially reduced, or insufficiently capped particles that are more susceptible to oxidation and agglomeration. Hence, the synthesis variables directly control the structural and chemical integrity of NPs throughout their storage and applications.

NPs are sensitive to storage conditions, such as temperature and light [13]. Exposure to ultraviolet (UV) or visible light wavelengths can trigger photochemical redox reactions, destabilise surface ligands, and accelerate aggregation or colour changes [14]. Elevated temperatures increase NP motion and diffusion, which increases the likelihood of collisions and coalescence [15], whereas low temperatures slow kinetic processes and prolong dispersion stability [16]. Storage media containing salts, proteins, or organic molecules further complicate stability through ligand exchange or ion bridging. For these reasons, NPs are best preserved under refrigerated and dark conditions that limit redox reactions and maintain surface passivation.

Despite extensive research on the synthesis of silver and selenium NPs, most stability assessments are limited to periods shorter than six months, and their long-term physicochemical behaviour remains poorly understood. These multicomponent systems comprise a metal or metalloid core, surrounded by a capping molecule, each of which may change over time. For example, when citrate-capped AgNPs were assessed over 104 days, some particles remained stable, while others aggregated [17]. Another study reported that *Lycium barbarum* polysaccharide–protein-stabilised SeNPs were stable for 40 days at 4 °C [18]. Therefore, the present study aimed to evaluate the long-term (12-month) physicochemical stability of synthesised silver and selenium NPs under controlled storage conditions, to identify factors that affect their structural integrity and functional performance.

## 2. Materials and Methods

### 2.1. Preparation of NPs

AgNPs and SeNPs were synthesised following the procedures described previously [19]. Briefly, chemical reduction of ionic silver and selenium precursors, namely silver nitrate (AgNO_3_) and sodium selenite (Na_2_SeO_3_), was performed. Several naturally sourced capping agents were incorporated into each NP formulation, including chitosan, casein, bovine serum albumin (BSA), and citrate, the latter with/without the addition of tannic acid. The NP suspensions were centrifuged, washed and the resuspended NPs were stored in sterile light-proof vials at 4 °C for 12 months. Immediately after synthesis, the suspensions were characterised to determine their physicochemical properties, as detailed previously [19]. The same batch of NPs were subsequently maintained for 12 months, then characterised using the same analytical procedures, including Tyndall light scattering, UV–visible spectroscopy, transmission electron microscopy (TEM) and inductively coupled plasma optical emission spectroscopy (ICP–OES). Their respective formulation codes, capping agents, synthesis approaches, initial mean particle sizes, and elemental concentrations are summarised in Table 1.

### 2.2. Characterisation

Tyndall scattering: The Tyndall effect was used as an in situ qualitative check to confirm NP formation in colloidal suspensions, following the approach described by Almuqrin et al. (2025) [19]. In short, a parallel visible laser beam was directed through a 1 mL aliquot of each NP suspension placed in a quartz cuvette. Scattering of the light path indicated the presence of dispersed NPs. Three handheld laser pointers of different wavelengths (violet 405 nm, green 532 nm, and red 670 nm; <5 mW, class IIIa) (Memory Tech, Sydney, NSW, Australia) were utilised to visualise this scattering. Deionised (DI) water served as the negative control. Images were captured in a dark room using a smartphone camera (model: iPhone 15 Pro; Apple Inc., Cupertino, CA, USA) positioned at right angles to the laser path. Safety procedures for class IIIa lasers, including the use of protective goggles and fixed beam alignment, were followed. This qualitative test was performed at baseline and after storage for 12 months.

UV–Vis spectroscopy: Absorbance data were obtained using a spectrophotometer (GENESYS™ 10S UV–Vis spectrophotometer, Thermo Fisher Scientific, Waltham, MA, USA). Prior to analysis, samples underwent vortex mixing followed by ultrasonication for 5 min. DI water was used as a control.

TEM: NP size distribution and morphology were analysed using TEM, following the protocol described in the synthesis study [19]. TEM imaging was conducted in the Central Analytical Research Facility (CARF) of the Queensland University of Technology (QUT). NP suspensions were vortex mixed then ultrasonicated for 5 min to achieve uniform dispersion before being drop-cast onto 300-mesh lacey formvar/carbon-coated copper grids. Samples were examined using a JEOL JEM-2100 TEM (JEOL Ltd., Tokyo, Japan) operating at 200 kV, and micrographs were captured with a 4 K TVIPS CCD camera. Image analysis and particle size measurements were performed in ImageJ (version 1.53m, NIH, Bethesda, MD, USA) using at least 100 nanoparticles to obtain representative data. TEM imaging was conducted at baseline and at 12 months for the same batch to assess changes in particle morphology during long-term storage.

For each NP formulation, one independent synthesis batch (*n* = 1) was evaluated at baseline and after 12 months of storage. TEM measurements of at least 100 individual particles per formulation were used to describe particle size distributions and were not considered independent experimental replicates.

ICPOES: The elemental composition of the nanosuspensions was analysed using a PerkinElmer 8300DV ICP-OES system (Waltham, MA, USA) equipped with an ESI SC-4DX autosampler and a PrepFAST 2 sample handling unit for online internal standardisation and automated dilution, as described previously [19]. Prior to analysis, the nanosuspensions were vortexed and ultrasonicated to facilitate the redispersion of settled or aggregated nanoparticles and promote sample homogeneity. Subsequently, the samples were diluted 1:10 in 2% ultra-pure nitric acid. Each sample was analysed in triplicate (technical replicates). Instrument calibration was performed using external standardisation, with multi-element standards prepared in-house from ICP-MS-grade single-element stock solutions (High Purity Standards, Charleston, SC, USA). Sub-boiling distilled nitric acid was used to prepare all calibration standards and blank solutions. A combination of axial and radial viewing modes was employed, with the radial mode specifically used for alkali metals (Li, Na, and K). Method robustness, accuracy, and precision were assessed through continuous analysis of multiple certified reference materials (CRMs) covering a range of matrices and analyte concentrations (National Institute of Standards and Technology, Gaithersburg, MD, USA; United States Geological Survey, Reston, VA, USA).

The use of ICP-OES enabled accurate quantification of the NPs’ elemental content without altering the organic capping layers, ensuring that the concentration measurements reflected true metal/metalloid levels, independent of the stabilising agents. Post storage analyses were performed to detect any compositional changes indicative of NP degradation or elemental leaching.

## 3. Results

**Figure 1:**
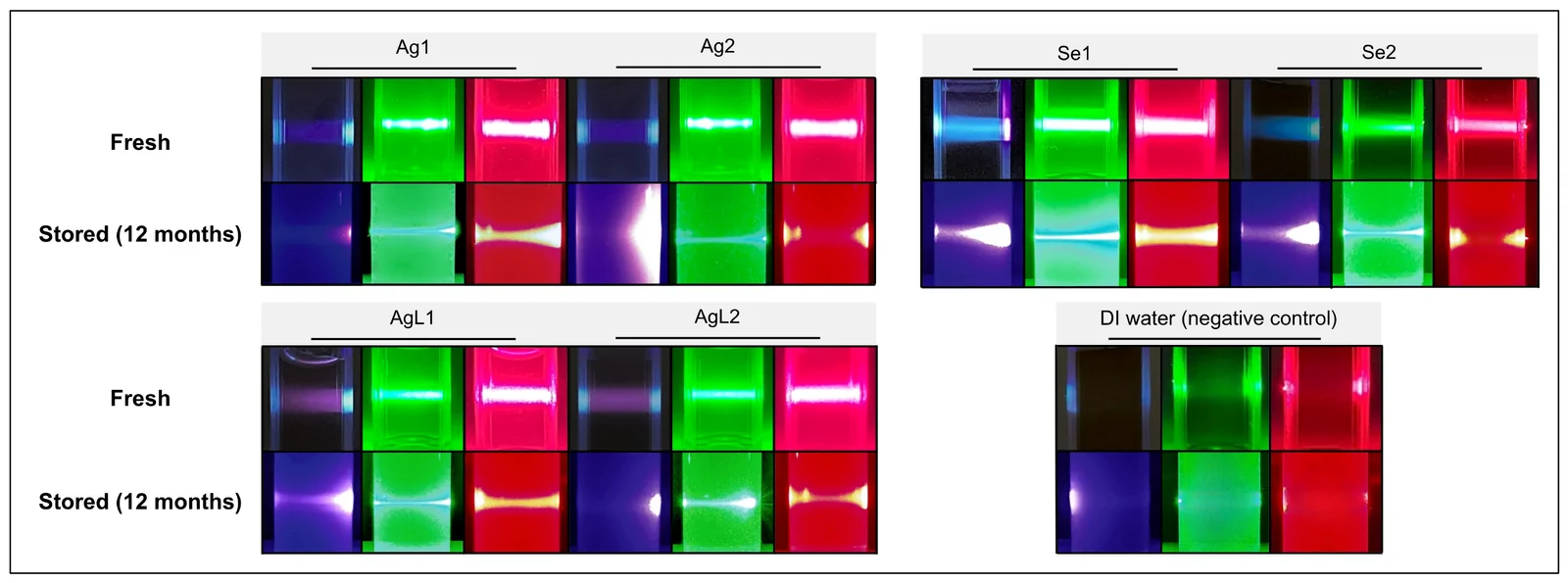
A collage of Tyndall scattering images obtained immediately after synthesis and after 12 months of storage.

Tyndall scattering: In general, the stored samples exhibited less distinct light scattering compared to their freshly prepared counterparts, as shown in Figure 1. However, visible Tyndall scattering remained observable after a year of storage, particularly in the citrate capped silver and selenium formulations, both of which showed strong, well-defined bands. On the contrary, in Ag1 and Ag2, scatter of laser beams showed moderate brightness but with slightly discontinuities in the beam paths. The AgL2 suspension displayed weak and irregular Tyndall scattering. Under blue light illumination (405 nm), light scattering was faint to absent in most Ag1, Ag2, and AgL2 samples, but remained bright in AgL1 and in selenium NP suspensions.

**Figure 2:**
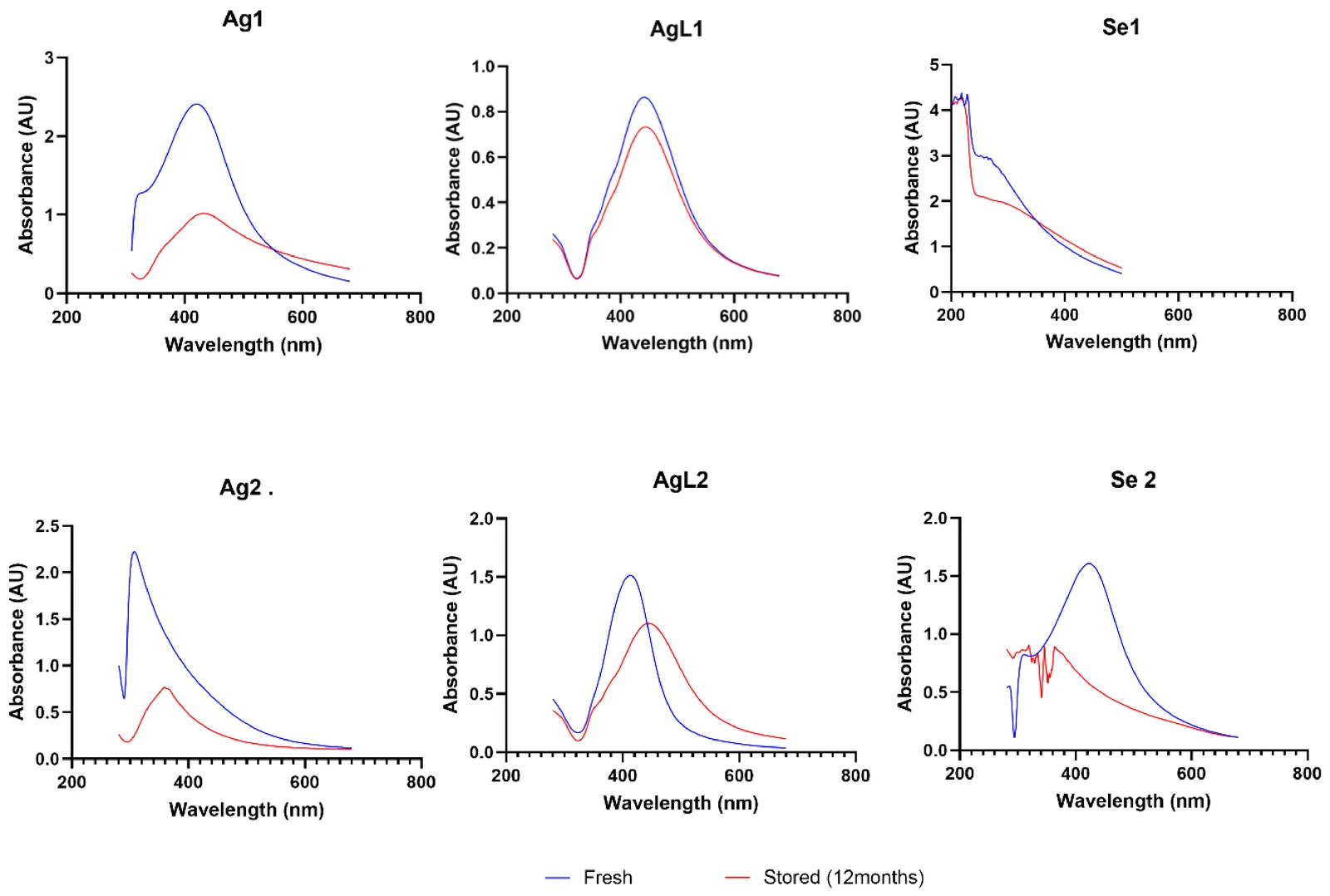
UV–visible light absorbance spectra of freshly synthesised and 12-month-old NP suspensions.

UV–Vis spectroscopy: As shown in Figure 2, after 12 months of storage, the absorbance decreased across all silver systems, accompanied by red shifts and band broadening. AgL1 retained the most well-defined and symmetric surface plasmon resonance (SPR) peak, with little reduction in intensity, while AgL2 underwent noticeable red shift of the peak absorbance, giving higher readings at longer wavelengths. Both Ag1 and Ag2 had lower absorbance readings after 12 months storage when compared to fresh samples.

With regard to the two aged SeNP samples, both showed substantial decreases in absorbance, particularly Se2.

Overall, the spectroscopic data indicate that all NP formulations experienced some degradation during storage. AgL1 demonstrated the greatest stability, maintaining the most consistent absorbance after 12 months.

**Figure 3:**
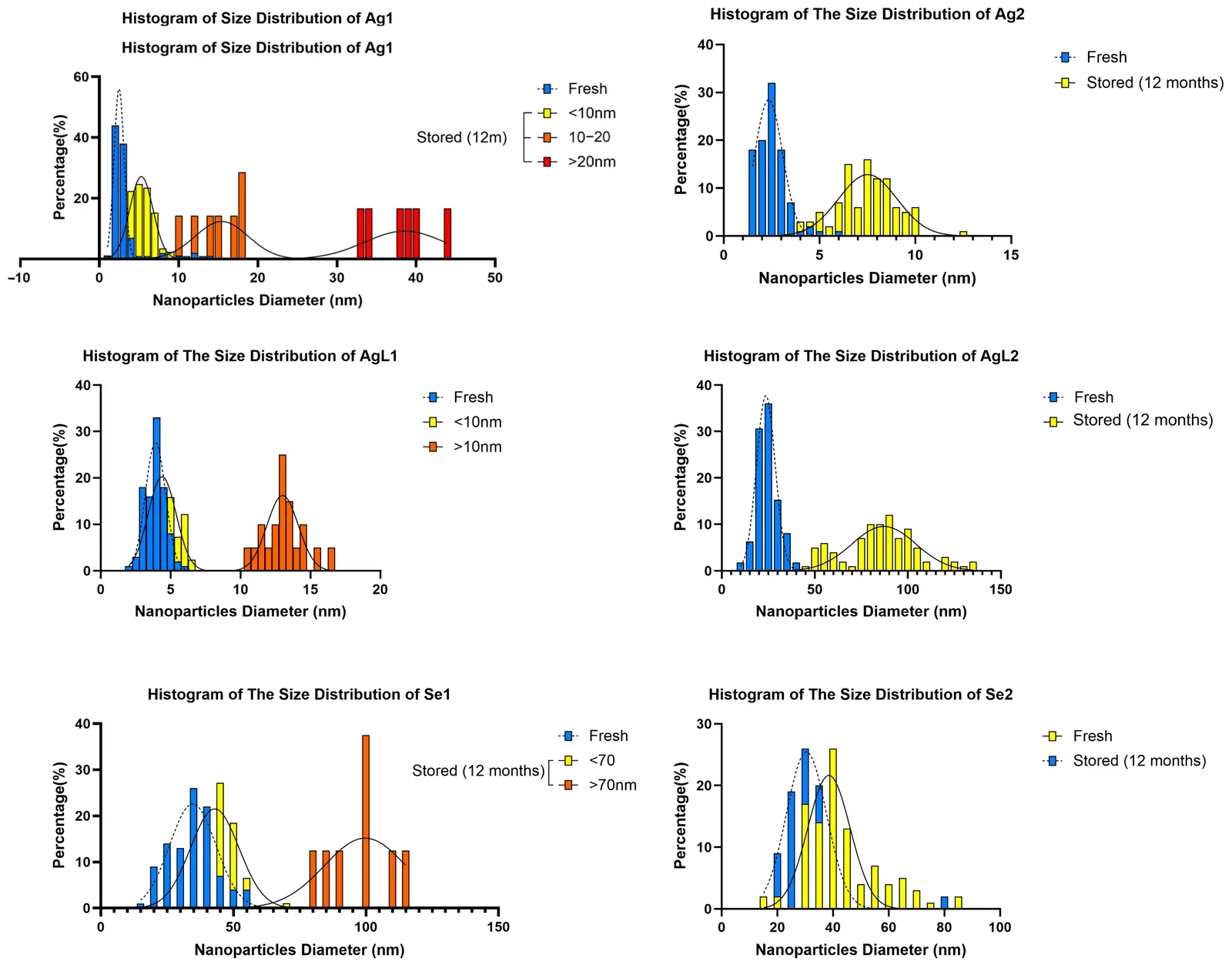
Size distribution histograms of the NPs measured by TEM immediately after synthesis and after 12 months of storage.

TEM: Particle size distribution analysis from TEM micrographs revealed obvious differences between fresh and stored NPs (Figure 3). In all silver formulations, the fresh samples (blue bars) exhibited narrow, monomodal distributions. After storage, the distributions shifted toward larger sizes, with increased polydispersity. Both Ag1 and Ag2 showed the formation of secondary size groups around 20–40 nm, while smaller NPs (<10 nm) decreased in proportion. The AgL1 formulation maintained the narrowest distribution, with most particles remaining below 15 nm even after 12 months. In contrast, AgL2 developed a significantly broader dual profile extending up to 100 nm. For the selenium NPs, both forms displayed similar trends. The fresh samples showed compact distributions centred below 50 nm, whereas the stored suspensions developed additional size categories beyond 70 nm. In Se1, where large aggregates were most evident, the overall size range shifted toward approximately 100 nm.

**Figure 4:**
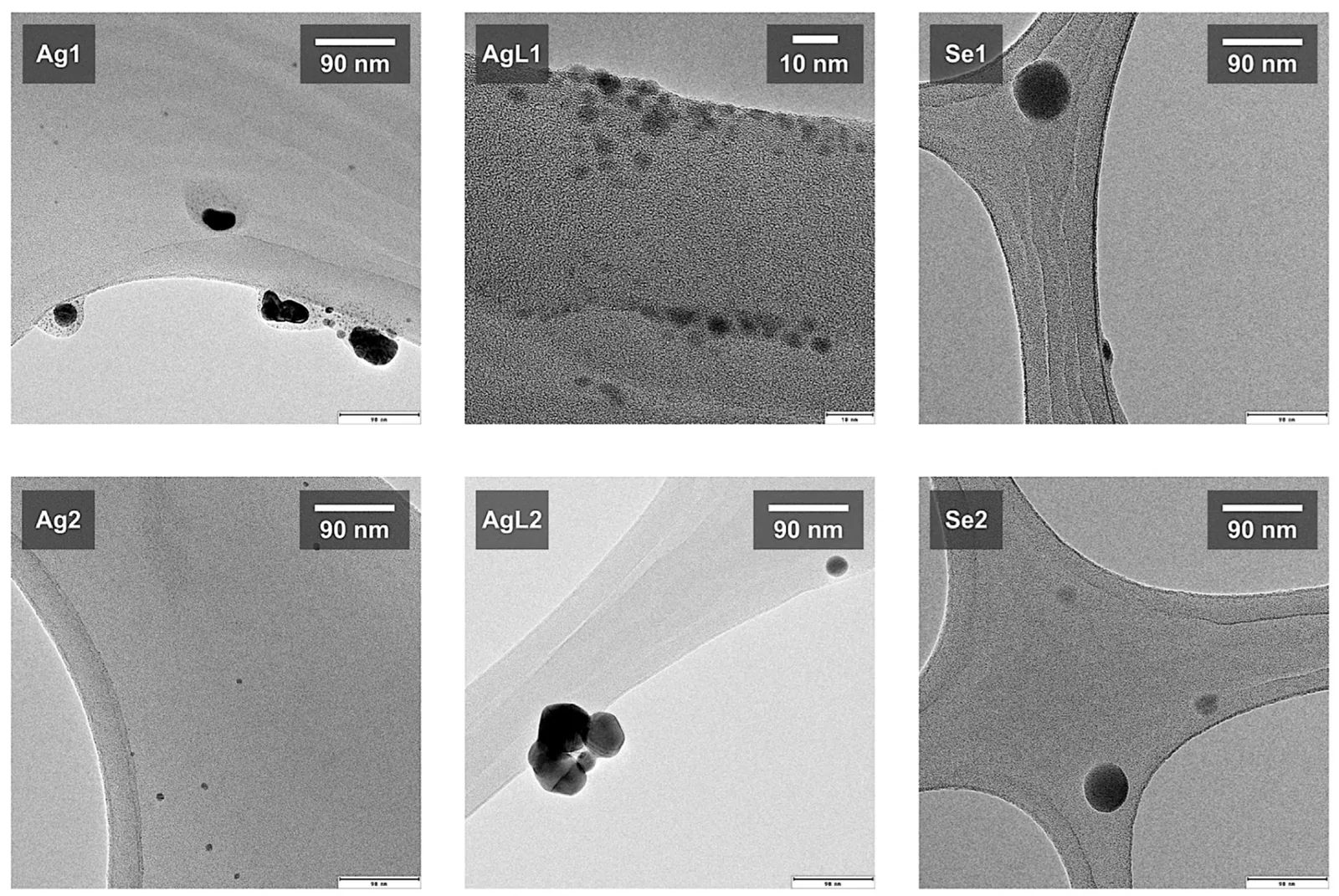
TEM micrographs of the synthesised NPs after 12 months of storage.

Based on the morphology of NPs under TEM imaging, spherical morphologies predominated, and this remained comparable in aged samples compared to fresh samples, except for Se1, which also exhibited elliptical or egg-shaped NPs (Figure 4). Moreover, irregular aggregates and clustered formations were observed in several samples, particularly those capped with casein and BSA.

ICP-OES: In general, elemental concentrations were reduced at 12 months. The decline was marked in AgL2, Se1, and Se2, moderate in Ag1 and Ag2, and minimal in AgL1. These data are shown in Table 2. Trace amounts of Cr, Fe, and Zn were present in both fresh and aged samples, consistent with minor background contamination from handling or equipment, rather than changes during storage. Their very low levels indicate no significant impact on sample integrity (Table 3).

## 4. Discussion

The present study evaluated the stability of silver and selenium NPs over a 12-month storage period. Characterisation procedures were identical to those used immediately after synthesis, with the same samples followed over time [19]. The tested NPs showed varying degrees of instability, such as the formation of large aggregates, altered light absorbance, and reduced elemental concentration. These were particularly marked in the selenium NP formulations.

Within the silver NPs, the citrate-capped AgL1 showed the best preservation of physicochemical characteristics over 12 months. This can be explained by its non-peptide nature, making it immune to proteolysis, and to robust surface protection of the NPs by combining electrostatic and steric stabilisation. The citrate anions impart a prominent negative charge through which particles repel each other and remain well dispersed [20]. Past work has shown that the stability of silver NPs capped with citrate is influenced by ionic strength [21], pH [22], and light exposure [17]. Other studies have shown that citrate-capped AgNPs remain stable only for short durations (≤7 days) in low-ionic-strength media, but readily aggregate in saline or high-salt environments [23]. In another work, Gorham et al. (2014) found that AgNPs stabilised with citrate gradually lose integrity through oxidation and photoreduction unless stored in the dark, in deoxygenated conditions, and at high concentrations with a high concentration of citrate [17]. Furthermore, in saline or phosphate-containing media, silver NPs capped with citrate aggregate faster than silver NPs with polyvinylpyrrolidone PVP [24].

In the present study, AgL1, which was synthesised in ultra-pure DI water and stored in the dark, maintained consistent absorbance properties over time, as well as structural integrity and compositional properties, compared to other Ag NPs. Average particle size changed from a single peak at 4 nm to a bimodal distribution with peaks at 4 and 12 nm over the period of storage. This is consistent with a minor degree of aggregation. The superior performance of AgL1 may also reflect the synthesis method used, which was based on careful calculations of stoichiometry and was deliberately slow, and also avoided high temperatures. These differences in synthesis methods may explain the strong performance for the citrate-capped AgNPs in the current study compared to past work.

Compared to AgL1, other AgNPs demonstrated lower stability. Ag1 and Ag2 exhibited degradation across all metrics. While spectroscopic analysis indicated that both retained their absorbance peak (λmax), the peaks became flatter and higher readings were observed at longer light wavelengths. This can be explained by aggregation of initially ultra-small particles from the synthesis batches (3.6 ± 3.4 nm and 2.5 ± 0.76 nm, respectively) to form larger size clusters (14.83 ± 3.03 nm and 38.9 ± 3.9 nm in Ag1, and 7.4 ± 1.6 nm in Ag2).

The tannic acid-capped material AgL2 showed significant deterioration, evidenced by an 88.6% loss in concentration and distinct aggregation in TEM micrographs. The average particle size increased from 24.17 ± 5.42 nm to 79.03 ± 27.03 nm (Table A1).

That variations in capping agent alter stability is to be expected. In a previous study, casein-coated AgNPs were colloidally stable for 9 months, based on absorbance data [25]. Likewise, Martin et al. (2014) [26] showed that a corona of BSA stabilises AgNPs in acidic conditions, but once that protein layer is disrupted, the silver core becomes exposed, leading to rapid oxidative dissolution and agglomeration of NPs [26]. Using in-situ ultra-small angle X-ray scattering (USAXS), they found that after a breach of the BSA coating, dissolution followed second-order kinetics, while TEM revealed that localised protein shell rupture preceded complete particle loss.

The key takeaway is that coating integrity as well as coating type controls the longevity of AgNPs. With AgL2, the rapid synthesis method used a one-step co-reduction route, and likely produced a heterogeneous, weakly bound ligand shell. In contrast, AgL1, which was prepared by the gradual, dropwise introduction of citrate, allowed for more uniform nucleation and more consistent surface capping, forming a compact electrostatic barrier that was superior for preserving NP integrity.

Selenium NPs showed poor stability, degrading far more rapidly than silver NPs. Over time, their spectroscopic absorbance peak collapsed, while TEM images showed that the particles had agglomerated (reaching sizes beyond 70–100 nm). These observations, coupled with the ICP–OES finding of >99% elemental selenium reduction from the colloid, indicate that the SeNPs aggregated quickly, or transformed into ionic or non-dispersed forms. This finding aligns with past reports that SeNP colloids have low stability, often losing their nanoscale dispersion and functional properties within days to weeks if not vigorously stabilised [27]. Uncapped or insufficiently stabilised SeNPs are susceptible to aggregation and precipitation during storage, whereas surface functionalisation with polymers or polysaccharides can improve colloidal stability and reduce particle aggregation [28]. The chemical nature of elemental selenium underlies this instability. Unlike noble metals, selenium is more reactive and can undergo gradual oxidation or allotropic conversion. Amorphous SeNPs tend to transform into larger crystalline (trigonal) selenium over time, a process that induces particle growth, shape change, and loss of colloidal stability [29].

The stability of NPs is a critical determinant of their clinical translation as it influences shelf life and formulation logistics, as well as bioavailability, therapeutic efficacy, and toxicity. Unstable NPs, such as rapidly degrading SeNPs that aggregate into micron-scale particulates, may trigger immunogenic or foreign body responses. Moreover, degradation during storage may lead to inconsistent dosing, reduced biological activity, or the formation of byproducts, which could affect safety. In contrast, formulations with more robust physicochemical stability, such as AgL1, are more likely to retain consistent pharmacological behaviour over time, and to meet regulatory standards for quality control. Therefore, optimising NP stability is not only a formulation concern but a prerequisite for safe, scalable clinical use.

The present study has several limitations. The stability assessments were limited to 12 months of storage at a single temperature, and did not include accelerated aging at higher temperatures or in vivo pharmacokinetic profiling. Additionally, while elemental loss and aggregation were quantified, mechanistic studies on degradation pathways (e.g., oxidation state transitions or crystallisation) were not performed. Future studies should evaluate long-term stability under varied conditions, incorporate advanced surface characterisation (e.g., XPS, XRD), and assess the functional performance of NPs in biological models to better predict clinical behaviour and to guide formulation optimisation.

Another important limitation of this study is that each NP formulation was evaluated using a single independent synthesis batch (*n* = 1). Although at least 100 particles were analysed by TEM to characterise particle size distributions, these measurements represent individual particle observations rather than independent experimental replicates. Consequently, the findings are primarily descriptive, and inferential statistical analyses were not performed to assess differences between baseline and 12-month measurements. The absence of independent synthesis and storage replicates also limits the assessment of batch-to-batch variability and the generalisability of the observed stability trends. Future studies should incorporate multiple independently synthesised batches and storage replicates to enable statistical comparisons and provide more robust evidence of long-term formulation stability.

Although trace amounts of Cr, Fe, and Zn were detected, their potential contribution to NP oxidation and ageing was not evaluated. Future studies should investigate whether these trace impurities contribute to changes in NP stability during prolonged storage.

Ultrasonic pre-treatment before UV–Vis analysis may have disrupted particle agglomerates, potentially overestimating colloidal stability. The absence of non-sonicated controls is therefore a limitation of this study. On the other hand, the absence of DLS, PDI, pH, and zeta-potential measurements limited the assessment of hydrodynamic particle size, dispersity, and surface charge. Future studies should incorporate these techniques to provide a more comprehensive evaluation of colloidal stability.

Regarding significant elemental loss in the tested samples, ICP-OES cannot distinguish between dissolved ions and nanoparticle-associated elements; therefore, the observed reductions in elemental concentrations cannot be attributed to a specific mechanism. Future studies should incorporate ultrafiltration followed by separate elemental analysis of dissolved and particulate fractions to clarify whether these reductions result from sedimentation, dissolution, or phase transformation.

Although the observed physicochemical changes provide preliminary insights into formulation stability during prolonged storage, these findings should not be interpreted as confirmation of a defined shelf-life or retention of antimicrobial efficacy.

## 5. Conclusions

SeNPs exhibited the lowest physicochemical stability among all formulations tested. Rapid aggregation, substantial reductions in measured elemental concentrations, and the collapse of spectral features during storage at 4 °C under light-protected conditions highlighted their vulnerability. In contrast, AgNPs, specifically citrate-capped NPs, maintained superior structural integrity under the same conditions. These findings indicate that improving synthesis methods, capping agents, and storage conditions is crucial to prolong NP stability. However, the findings are limited to a single storage condition, and post-storage antimicrobial activity was not assessed. Future studies should investigate different storage conditions and functional stability to establish clinical applicability.

## Figures and Tables

**Table 1:** Summary of NP formulations and their initial physicochemical characteristics.

Formulation	Capping Agent	Initial Mean Particle Size (nm)	Initial Elemental Concentration (mg/L)
Ag1	Casein	3.59	57.44
Ag2	Bovine serum albumin (BSA)	2.51	265.8
AgL1	Citrate	3.90	121.6
AgL2	Citrate and tannic acid	24.17	119.2
Se1	Chitosan	34.87	683.1
Se2	Bovine serum albumin (BSA)	43.23	642.8

**Table 2:** Elemental analysis of NPs immediately after synthesis and after 12 months of storage.

Analyte	Ag1-Fresh	Ag1-Stored	Ag2-Fresh	Ag2-Stored	AgL1-Fresh	AgL1-Stored	AgL2-Fresh	AgL2-Stored	Se1-Fresh	Se1-Stored	Se2-Fresh	Se2-Stored	Limit of Reporting (mg/L)
	Conc. mg/L	Conc. mg/L	Conc. mg/L	Conc. mg/L	Conc. mg/L	Conc. mg/L	Conc. mg/L	Conc. mg/L	Conc. mg/L	Conc. mg/L	Conc. mg/L	Conc. mg/L	
**Ag**	57.44	41.99	265.8	120	121.6	113.4	119.2	13.64	0.678	0.4119	0.113	0.0861	0.050
**Al**	<LOR	<LOR	0.0761	<LOR	0.0728	<LOR	0.0718	<LOR	0.0707	0.0643	0.069	0.055	0.050
**B**	<LOR	<LOR	<LOR	0.0889	<LOR	0.0535	<LOR	<LOR	159	165.5	162	195.34	0.050
**Ca**	<LOR	0.107	0.0796	0.1569	1.569	0.2573	1.321	0.3486	2.706	3.199	0.1536	0.1622	0.050
**Cr**	<LOR	<LOR	<LOR	<LOR	<LOR	<LOR	<LOR	<LOR	0.0519	<LOR	<LOR	<LOR	0.050
**Fe**	<LOR	<LOR	<LOR	<LOR	<LOR	<LOR	<LOR	<LOR	0.2791	0.2765	<LOR	<LOR	0.050
**K**	3.913	0.5143	<LOR	<LOR	<LOR	<LOR	<LOR	0.5291	<LOR	<LOR	<LOR	0.35	0.250
**Mg**	<LOR	<LOR	<LOR	<LOR	<LOR	<LOR	<LOR	<LOR	2.774	2.931	0.9465	0.5334	0.250
**Na**	<LOR	0.6143	111	109.1	66.07	46.22	81.34	7.798	658.8	641	675.9	283.7	0.250
**S**	<LOR	<LOR	16.9	<LOR	<LOR	<LOR	<LOR	<LOR	0.5252	644.2	42.16	205.5	0.500
**Se**	<LOR	<LOR	<LOR	<LOR	<LOR	<LOR	<LOR	<LOR	683.1	0.4541	642.8	0.2732	0.250
**Sr**	<LOR	<LOR	<LOR	<LOR	<LOR	<LOR	<LOR	<LOR	0.0169	<LOR	<LOR	<LOR	0.010

Ba, Be, Cd, Co, Cu, Li, Mn, Mo, Ni, P, Pb, Si, Ti, V, Zn, and Zr levels were below the LOR in all fresh and stored samples.

**Table 3:** Changes in elemental concentration in NPs.

Sample	Baseline Concentration (mg/L)	After 12 Months (mg/L)	% Reduction
**Ag1**	57.44	41.99	26.9%
**Ag2**	265.8	120.0	54.8%
**AgL1**	121.6	113.4	6.7%
**AgL2**	119.2	13.64	88.6%
**Se1**	683.1	0.454	99.9%
**Se2**	642.8	0.273	99.96%

## Data Availability

The datasets generated and/or analysed during the current study are available from the corresponding author on reasonable request.

## Appendix A

**Table A1:** TEM analysis of particle sizes for fresh and stored NPs.

NPs		Mean	Minimum	Maximum	Std. Deviation
**Ag1-Fresh**		3.594	1.486	14.35	3.407
**Ag1-Stored**	<10 nm	5.400	1.489	9.879	1.507
	10–20 nm	14.83	10.49	18.15	3.030
	>20 nm	38.00	32.93	43.58	3.899
**Ag2-Fresh**		2.509	1.518	5.972	0.7692
**Ag2-Stored**		7.372	3.242	12.58	1.623
**AgL1-Fresh**		3.9	1.998	5.921	0.697
**AgL1-Stored**	<10 nm	4.51	2.898	6.697	0.907
	>10 nm	13.082	10.312	16.42	0.333
**AgL2-Fresh**		24.173	11.296	38.402	5.449
**AgL2-Stored**		79.044	14.972	135.688	27.35
**Se1-Fresh**		34.873	15.761	56.267	8.522
**Se1-Stored**	<70 nm	41.06	17.954	70.525	9.496
	>70 nm	97.463	81.006	113.871	10.87
**Se2-Fresh**		43.234	15.462	87.331	13.273
**Se2-Stored**		33.921	15.196	85.412	12.842

## References

[1] Phan H.T., Haes A.J. (2019). What does nanoparticle stability mean?. J. Phys. Chem. C.

[2] Habibullah G., Viktorova J., Ulbrich P., Ruml T. (2022). Effect of the physicochemical changes in the antimicrobial durability of green synthesized silver nanoparticles during their long-term storage. RSC Adv..

[3] Bélteky P., Rónavári A., Igaz N., Szerencsés B., Tóth I.Y., Pfeiffer I., Kiricsi M., Kónya Z. (2019). Silver nanoparticles: Aggregation behavior in biorelevant conditions and its impact on biological activity. Int. J. Nanomed..

[4] Bhiri N., Masquelez N., Nasri M., Nasri R., Hajji M., Li S. (2025). Synthesis, characterization, and stability study of selenium nanoparticles coated with purified polysaccharides from Ononis natrix. Nanomaterials.

[5] Tayebi S.S., Dowdall N., Hoare T., Mhaskar P. (2025). Data-driven optimisation of nanoparticle size using the prediction reliability enhancing parameter (PREP). Nanoscale.

[6] Thanh N.T.K., Maclean N., Mahiddine S. (2014). Mechanisms of nucleation and growth of nanoparticles in solution. Chem. Rev..

[7] Khoramian R., Issakhov M., Pourafshary P., Gabdullin M., Sharipova A. (2024). Surface modification of nanoparticles for enhanced applicability of nanofluids in harsh reservoir conditions: A comprehensive review for improved oil recovery. Adv. Colloid Interface Sci..

[8] Anand S., Pandiyan Kuppusamy R.R., Padmanabhan P. (2020). Insight into the kinetically and thermodynamically controlled biosynthesis of silver nanoparticles. IET Nanobiotechnol..

[9] Loghina L., Kaderavkova A., Chylii M., Frumarova B., Svec P., Slang S., Vlcek M. (2020). The systematic study of the precursor ratio effect in the Cd–Zn–S quantum dot synthesis. CrystEngComm.

[10] Altammar K.A. (2023). A review on nanoparticles: Characteristics, synthesis, applications, and challenges. Front. Microbiol..

[11] Thai V.-P., Nguyen H.D., Saito N., Takahashi K., Sasaki T., Kikuchi T. (2022). Precise size control and functionalisation of gold nanoparticles synthesised by plasma–liquid interactions: Using carboxylic, amino, and thiol ligands. Nanoscale Adv..

[12] Zhou F., Peterson T., Fan Z., Wang S. (2023). The commonly used stabilisers for phytochemical-based nanoparticles: Stabilisation effects, mechanisms, and applications. Nutrients.

[13] Izak-Nau E., Huk A., Reidy B., Uggerud H., Vadset M., Eiden S., Voetz M., Himly M., Duschl A., Dusinska M. (2015). Impact of storage conditions and storage time on silver nanoparticles’ physicochemical properties and implications for their biological effects. RSC Adv..

[14] Grillet N., Manchon D., Cottancin E., Bertorelle F., Bonnet C., Broyer M., Lermé J., Pellarin M. (2013). Photo-oxidation of individual silver nanoparticles: A real-time tracking of optical and morphological changes. J. Phys. Chem. C.

[15] Lehtinen K.E.J., Zachariah M.R. (2002). Energy accumulation in nanoparticle collision and coalescence processes. J. Aerosol Sci..

[16] Wang Y., Quinsaat J.E.Q., Ono T., Maeki M., Tokeshi M., Isono T., Tajima K., Satoh T., Sato S.-i., Miura Y. (2020). Enhanced dispersion stability of gold nanoparticles by the physisorption of cyclic poly(ethylene glycol). Nat. Commun..

[17] Gorham J.M., Rohlfing A.B., Lippa K.A., MacCuspie R.I., Hemmati A., Holbrook R.D. (2014). Storage wars: How citrate-capped silver nanoparticle suspensions are affected by not-so-trivial decisions. J. Nanopart. Res..

[18] Zhang J., Ji T., Yang X., Liu G., Liang L., Liu X., Wen C., Ye Z., Wu M., Xu X. (2022). Properties of selenium nanoparticles stabilised by Lycium barbarum polysaccharide–protein conjugates obtained with subcritical water. Int. J. Biol. Macromol..

[19] Almuqrin A., Seneviratne C.J., Walsh L.J., Zafar S. (2025). Tailored synthesis and profiling of capped silver and selenium nanoparticles for topical applications in paediatric dentistry. Dent. J..

[20] Henglein A., Giersig M. (1999). Formation of colloidal silver nanoparticles: Capping action of citrate. J. Phys. Chem. B.

[21] Tejamaya M., Römer I., Merrifield R.C., Lead J.R. (2012). Stability of citrate, PVP, and PEG-coated silver nanoparticles in ecotoxicology media. Environ. Sci. Technol..

[22] Prathna T.C., Chandrasekaran N., Mukherjee A. (2011). Studies on aggregation behaviour of silver nanoparticles in aqueous matrices: Effect of surface functionalisation and matrix composition. Colloids Surf. A.

[23] Park K., Tuttle G., Sinche F., Harper S.L. (2013). Stability of citrate-capped silver nanoparticles in exposure media and their effects on the development of embryonic zebrafish (Danio rerio). Arch. Pharm. Res..

[24] Ferreira A.M., Vikulina A., Loughlin M., Volodkin D. (2023). How similar is the antibacterial activity of silver nanoparticles coated with different capping agents?. RSC Adv..

[25] Bora A., Mishra P. (2019). Casein and Ag nanoparticles: Synthesis, characterization, and their application in biopolymer-based bilayer film. J. Food Process. Preserv..

[26] Martin M.N., Allen A.J., MacCuspie R.I., Hackley V.A. (2014). Dissolution, agglomerate morphology, and stability limits of protein-coated silver nanoparticles. Langmuir.

[27] Bužková A., Hochvaldová L., Večeřová R., Malina T., Petr M., Kašlík J., Kvítek L., Kolář M., Panáček A., Prucek R. (2025). Selenium nanoparticles: Influence of reducing agents on particle stability and antibacterial activity at biogenic concentrations. Nanoscale.

[28] Bai K., Hong B., He J., Hong Z., Tan R. (2017). Preparation and antioxidant properties of selenium nanoparticles-loaded chitosan microspheres. Int. J. Nanomed..

[29] Srivastava N., Mukhopadhyay M. (2013). Biosynthesis and structural characterisation of selenium nanoparticles mediated by Zooglea ramigera. Powder Technol..

